# The Receptor for Advanced Glycation Endproducts Does Not Contribute to Pathology in a Mouse Mesenteric Ischemia/Reperfusion-Induced Injury Model

**DOI:** 10.3389/fimmu.2015.00614

**Published:** 2015-12-07

**Authors:** Mike C. L. Wu, Timothy D. Gilmour, Susanna Mantovani, Trent M. Woodruff

**Affiliations:** ^1^School of Biomedical Sciences, The University of Queensland, Brisbane, QLD, Australia

**Keywords:** ischemia–reperfusion injury, RAGE, HMGB-1, C3a, neutrophils, small intestine

## Abstract

The receptor for advanced glycation endproducts (RAGE) can engage a diverse class of ligands and contribute to the immune and inflammatory response to infection and injury. It is known to be a pathogenic receptor in many inflammatory diseases, including ischemia/reperfusion (IR) injuries in several tissues; however, its role has not been investigated in IR injuries of the intestine to date. Mesenteric (or intestinal) IR leads to recruitment of inflammatory cells into intestinal interstitial spaces, which markedly disrupts intestinal mucosa. IR-induced mucosal injury is accompanied by the development of a local and systemic inflammatory response and remote organ injury, and results in high mortality in the clinic. We hypothesized that elimination of RAGE signaling using RAGE^−/−^ mice would result in decreased local and remote organ injury and reduced inflammation in a mesenteric IR model, and thus be a target for therapeutic intervention. We found that RAGE ligands including HMGB-1 and C3a were elevated after mesenteric IR indicating the potential for enhanced RAGE activation in this model. However despite this, wild-type and RAGE^−/−^ mice both displayed similar degrees of mesenteric injury, neutrophil infiltration, intestinal edema, cytokine generation, neutrophil mobilization, and remote organ injury after mesenteric IR. We, therefore, conclude that despite its role in other organ IR injuries, and the robust production of RAGE ligands after intestinal ischemia, RAGE itself does not directly influence tissue injury and the inflammatory response in mesenteric IR.

## Introduction

The gastrointestinal tract takes up a significant portion of individual circulating blood, and a decrease in blood supply to the intestine even for a short period due to systemic hypotension, major cardiovascular surgery or trauma, can lead to intestinal ischemia (1). Delayed presentation of intestinal ischemia renders the in-hospital mortality rate still above 60% (2). Clinicians also encounter the problems associated with the salvage of ischemic gut, where reperfusion leads to a local and systemic inflammatory response that exacerbates tissue destruction and other complications, termed ischemia/reperfusion (IR) injuries (3).

The receptor for advance glycation endproducts (RAGE) is a transmembrane innate immune receptor that upon ligation can induce a variety of inflammatory responses and oxidative stress (4, 5). RAGE is capable of interacting with several ligands, including its namesake glycoprotein advanced glycation endproducts (AGE), high-mobility group protein box 1 (HMGB-1), S100 proteins, and complement fragment 3a (C3a) (6, 7). Given the large number of RAGE ligands associated with the inflammatory response, activation of this receptor has a critical impact on facilitating chronic diseases via chronic inflammation, for example diabetic conditions and Alzheimer’s disease (8, 9). It is also strongly implicated in the pathogenesis of IR injuries, with several studies demonstrating genetic deficiency or inhibition of RAGE to be protective in IR injuries to the heart, liver, lung, and brain (10–13). To date, however, no studies have addressed whether RAGE is pathogenic during the development of intestinal IR injury.

Intestinal IR injuries in the mouse lead to a variety of pathological sequela including mobilization and recruitment of neutrophils, oxidative damage, edema, production of inflammatory cytokines, and systemic inflammation leading to remote organ injury (14). Given that RAGE activation can contribute to many of these same pathways and inhibition of RAGE is protective in IR injuries of other organs, this suggests that RAGE blockade could be a therapeutic target for treating intestinal IR injuries. In this study, we therefore aimed to determine the pathogenic role of RAGE in intestinal IR injury. We first measured RAGE ligand levels after mesenteric IR and then measured mucosal damage in RAGE deficient (RAGE^−/−^) mice. In addition, inflammatory cells, inflammatory mediators, and injury markers were also measured. Interestingly, we found that despite an upregulation of RAGE ligands following mesenteric IR, deficiency of RAGE did not impact on the disease pathology, suggesting other signaling pathways predominate in this model.

## Materials and Methods

### Animals

Male C57BL/6 wild-type (WT) mice and RAGE signaling-deficient mice (RAGE^−/−^) on a C57BL/6J genetic background were maintained at the University of Queensland’s Biological Resources Animal Facilities under specific pathogen free conditions. Male mice aged 10–12 weeks weighing 20–25 g were selected for all experiments. Homozygous RAGE^−/−^ mice have the extracellular domain of RAGE (exons 2–7) removed and thus express a non-functional RAGE protein (15, 16). The experimental protocols were approved by the University of Queensland’s Animal Ethics Committee.

### Intestinal Ischemia Reperfusion Injury Model

Mice were anesthetized by a ventilated system containing 4% of isoflurane with oxygen supply (2 L/min) during the surgical operation. To induce mesenteric IR, the superior mesenteric artery (SMA) was exposed and occluded with a loop ligature for 30 min to induce non-traumatic intestinal ischemia as previously described (17, 18). The ligature was then removed to allow intestinal tissue reperfusion for 150 min, which results in moderate–severe intestinal damage (18). Both WT and RAGE^−/−^ mice underwent IR surgery. To establish a baseline, sham-operated WT mice underwent the same surgical procedures, but the SMA was not occluded. Following the reperfusion phase, mice were then euthanatized, and pieces of intestinal tissue from the distal jejunum/early ileum and whole blood [collected in 1 mg/mL EDTA and 0.1 mg/mL nafamostat mesylate (19)] were obtained for other analyses described below.

### Histology and Tissue Injury Evaluation

Tissues were fixed in 4% paraformaldehyde (Sigma-Aldrich, USA) for a minimum of 72 h and embedded in paraffin. Tissue sections (6 μm) were stained with hematoxylin and eosin (H&E) by following a standard H&E staining protocol. Tissue injury score was determined in a blinded fashion using a graded scale adapted from Chiu et al. (20).

### Wet/Dry Weight

A portion of the intestine (5–10 cm) was weighed to obtain wet weight (g). The tissue was allowed to dry for 48 h at 80°C and weighed for dry weight. Wet/dry weight ratio, as a measure of edema, was calculated by the formula: wet weight (g)/dry weight (g).

### Myeloperoxidase Level

Neutrophil accumulation and activation in the tissue was quantitated by the level of myeloperoxidase enzyme in the tissue. A portion of intestine was homogenized and sonicated in phosphate buffered saline (PBS; pH 6.0) containing 1% protease inhibitor cocktail (Merck-Millipore, USA) and 0.5% Hta-Br (Sigma-Aldrich, USA). The homogenate was incubated with the substrate containing 2.85 mg/mL *O*-dianisidine and 2.3% hydrogen peroxide in deionized water. The absorbance of the sample was then measured spectrophotometrically at 460 nm at 15 min and normalized to the total protein level of the homogenate measured by BCA protein Assay (Thermo Fisher Scientific, Australia).

### Intestinal Neutrophil Staining (Leder’s Stain)

Neutrophils in the intestine were stained using a naphthol AS-D chloroacetate esterase cytochemical staining kit (Sigma-Aldrich, USA), which identifies specific leukocyte esterases predominantly expressed in granulocytic neutrophils. Harvested tissues were fixed in 4% paraformaldehyde, embedded in paraffin and, microtome sectioned and then stained with naphthol esterase solution, according to the manufacturer’s protocol. Cells with bright-red granulations in the mucosa were counted in a blinded method and expressed as the number of neutrophils per villus.

### Leukocyte and Neutrophil Count

EDTA blood (1 mg/mL) was collected from the inferior vena-cava and centrifuged. White blood cells were isolated by lysing erythrocytes with red blood cell lysis solution (0.85% ammonium chloride in PBS) followed by hypotonic shock (21). Total leukocyte numbers were determined by using an up-right light microscopy and a hematocytometer. The neutrophil population (%) was identified and counted in triplicate blood smears stained by Diff-Quik staining kit (Thermo Fisher Scientific, Australia). Circulating neutrophil counts were calculated by multiplying total white blood cell count (cells per millilitre) to neutrophil population (%).

### Alanine Transaminase and Alkaline Phosphatase

Liver injury was quantified by measuring alanine transaminase (ALT) and alkaline phosphatase (ALP) levels in the plasma as previously described (22) and according to the manufacturer’s instructions (ALT and ALP reagents; Thermo Fisher Scientific, Australia).

### Inflammatory Protein Levels

Plasma cytokines, HMGB-1, and IL-6 and complement factor C3a levels were measured by enzyme-linked immunosorbent (ELISA) kits (Chondrex, USA; BD Biosciences, Australia), according to their manufacturers’ instructions. The concentrations of these proteins for the samples were calculated by using a linear regression analysis of their correspondent standard curves.

### Statistics

All experimental results are expressed as mean ± SEM. Data analysis was performed using GraphPad Prism 6.0 software (GraphPad software, Inc., USA). Statistical comparisons were made using a one-way ANOVA with Dunnett’s post-test, or *t-*test (two-tailed distribution).

## Results

### Mesenteric IR Induces Release of Ligands for RAGE

**Figure 1:**
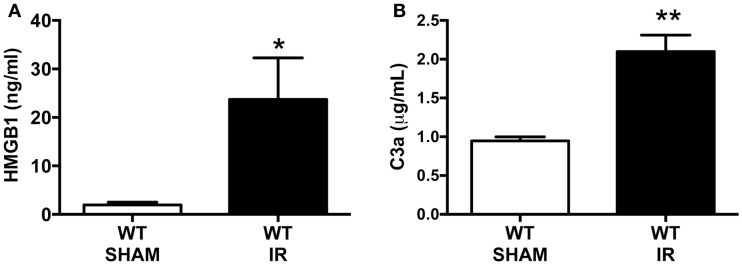
**Intestinal ischemia reperfusion (IR) injury induces systemic blood elevation of RAGE ligands**. Wild-type (WT) mice were subjected to 30 min mesenteric artery occlusion, followed by 150 min of reperfusion to induce intestinal IR injury. Alternatively, sham-operated mice underwent the same procedure, except the mesenteric artery was not occluded (SHAM). The plasma levels of **(A)** HMGB1 and **(B)** C3a were measured using standard ELISA kits at the end of the reperfusion period. Data are presented as mean ± SEM, *n* = 4 (WT-SHAM) *n* = 8 (WT-IR) where *p < 0.05, **p < 0.01 compared with WT-SHAM.

There are several molecules that are reported to interact with RAGE and that include the damage-associated molecular pattern (DAMP) molecule, secreted HMGB-1, and the complement activation fragment C3a, both of which are generated following tissue injury (6, 7). To assess whether our mouse model of IR injury generated these RAGE ligands, HMGB-1 and C3a systemic blood concentrations were measured by using standard ELISAs. The plasma concentration of HMGB-1 was dramatically increased by approximately 12-fold following IR, compared to sham-operated IR mice (Figure 1A). We also found a twofold increase in C3a plasma concentrations after IR compared to sham-operated mice (Figure 1B). These data show that the intestinal IR setting induced elevations of RAGE ligands in the circulation, and thus indicates the potential for enhanced RAGE activation after mesenteric IR.

### Elimination of RAGE Does Not Reduce Mucosal Injury Following Mesenteric IR

**Figure 2:**
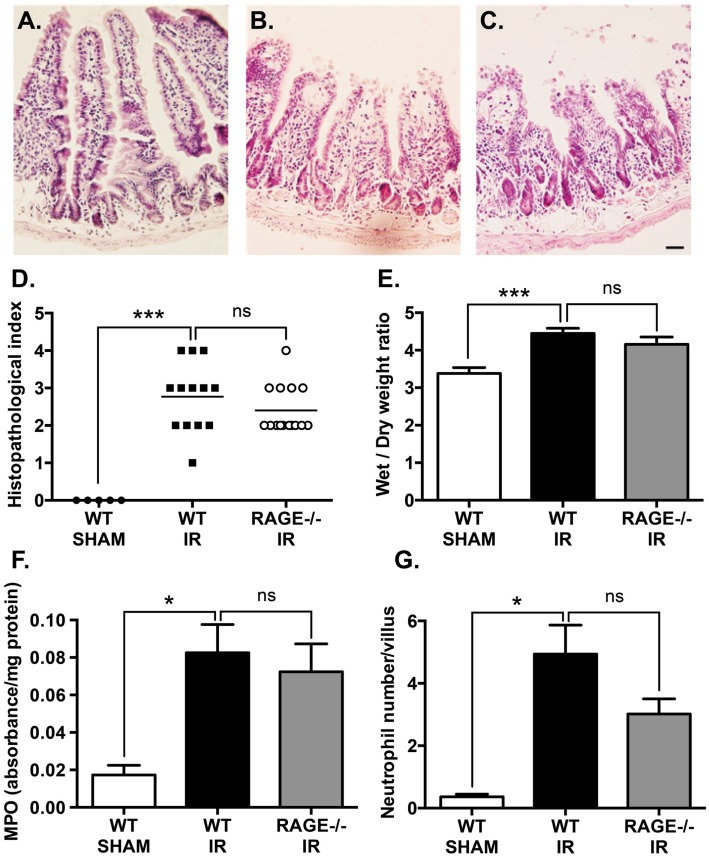
**Elimination of RAGE signaling does not impact mucosal damage and neutrophil accumulation following mesenteric IR**. Mice were subjected to 30 min mesenteric artery occlusion, followed by 150 min reperfusion. **(A–C)** Representative H&E staining of cross-sections of ileum from **(A)** sham-operated wild-type mice (WT-SHAM), **(B)** mesenteric IR wild-type mice (WT-IR), and **(C)** mesenteric IR RAGE^−/−^ mice (RAGE^−/−^ IR) groups. Scale bar = 50 μm. The mucosal injury was semi-quantitated as expressed as histopathological index **(D)** demonstrating no significant effect on IR injury following RAGE elimination. Increases in intestinal edema, as measured by wet/dry weight ratios **(E)**, were also not impacted by RAGE signaling elimination. Similar results were seen following quantification of myeolperoxidase levels in intestinal homogensates, normalized by total protein levels **(F)**, and esterase stained (Leder’s) neutrophil counts in the entire cross-section of intestine and normalized to number of villi **(G)**. Data are presented as mean ± SEM, *n* = 5 (WT-SHAM); *n* = 13 (WT-IR); *n* = 15 (RAGE^−/−^ IR) where *p < 0.05, ***p < 0.001, and ns = not-significant (*p* > 0.05).

Mesenteric IR induces a marked damage to intestinal mucosa. In our study, haemotoxylin and eosin staining demonstrated mesenteric IR-induced mucosal damage which included villus epithelial lifting/loss and *lamina propria* swelling (Figure 2A), compared sham-operation (Figure 2B). This mucosal damage caused by IR was not mitigated in RAGE deficient (RAGE^−/−^) mice undergoing IR (Figure 2C). Tissue damage was semi-quantitated by scoring tissue from individual mice in a blinded manner, demonstrating elimination of RAGE had no significant impact on intestinal injury scores after IR (Figure 2D).

Next, tissue edema was measured as an indicator of vascular leakage of a common pathological event following mucosal damage. Mesenteric IR induced an increase in intestinal wet/dry weight ratios, however, elimination of RAGE had no impact on the water content in the intestine after IR (Figure 2E).

Neutrophils are a key player of the villi destruction in intestinal IR injury (14). We examined neutrophil accumulation by staining intestinal sections for neutrophils and measuring myeloperoxidase levels in the IR-injured intestines (18). As expected, IR induced a marked increase in neutrophil accumulation in the injured intestine and increased myeloperoxidase levels; however, elimination of RAGE did not show any significant alteration in neutrophil accumulation after IR (Figures 2F,G).

### Elimination of RAGE Does Not Impact White Blood Cell and Neutrophil Mobilization Following Mesenteric IR

**Figure 3:**
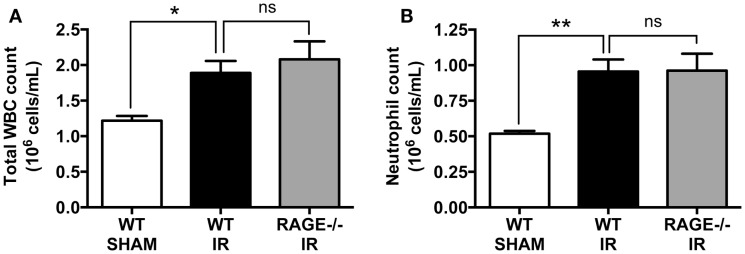
**Elimination of RAGE does not affect total white blood cell and blood neutrophil counts following intestinal IR**. Mice were subjected to 30 min mesenteric artery occlusion, followed by 150 min reperfusion. The numbers of **(A)** white blood cells (WBC) and **(B)** neutrophils, in the peripheral blood collected after 150 min reperfusion, demonstrating that RAGE does not influence IR-induced leukocyte or neutrophil mobilization. Data are presented as mean ± SEM, *n* = 5 (WT-SHAM); *n* = 13 (WT-IR); *n* = 15 (RAGE^−/−^ IR) where *p < 0.05, **p < 0.01, and ns = not-significant (*p* > 0.05).

We have previously shown that intestinal reperfusion is strongly accompanied by a rapid mobilization of leukocytes (predominantly neutrophils) from bone marrow reservoirs into the circulation (18). Furthermore, the degree of neutrophil mobilization is correlated to the degree of neutrophil infiltration into the reperfused intestine and subsequent intestinal injury, and thus a key disease parameter of the potential for IR injury (18). As in our prior studies, mesenteric IR induced increases in both circulating white blood cells (Figure 3A), which were chiefly neutrophils (Figure 3B). However, again as for mucosal injury markers, the numbers of mobilized leukocytes or neutrophils were not affected by eliminating RAGE signaling (Figures 3A,B).

### Elimination of RAGE Does Not Reduce Remote Organ (Liver) Injury and Systemic Inflammation Following Mesenteric IR

**Figure 4:**
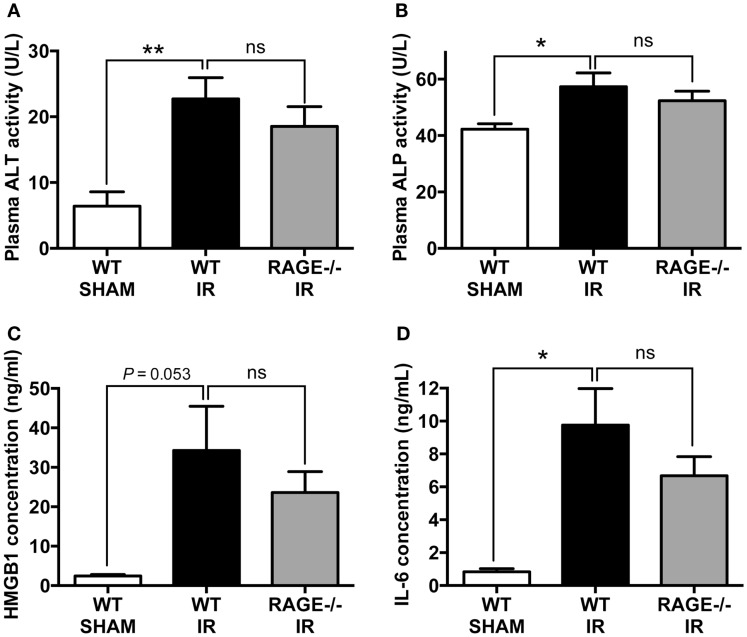
**Elimination of RAGE does not influence remote organ (liver) injury and systemic inflammation following intestinal IR**. Mice were subjected to 30 min mesenteric artery occlusion, followed by 150 min reperfusion. Peripheral blood samples were collected from WT-SHAM, WT-IR, and RAGE^−/−^ IR groups for enzyme activity and pro-inflammatory markers. **(A,B)** Activities of liver enzymes ALT **(A)** and ALP **(B)** measured as markers of liver injury. **(C,D)** Levels of pro-inflammatory mediators HMGB-1 **(C)** and IL-6 **(D)**. Data are presented as mean ± SEM, *n* = 5 (WT-SHAM); *n* = 13 (WT-IR); *n* = 15 (RAGE^−/−^ IR) where *p < 0.05, **p < 0.01, and ns = not-significant (*p* > 0.05).

Systemic inflammation and remote organ injury are often complications in intestinal IR injury (23, 24), which can lead to significant morbidity and mortality. We, therefore, finally examined liver injury markers, alanine aminotransferase (ALT), and ALP and pro-inflammatory mediators, including HMGB-1 and interleukin-6 (IL-6) in the peripheral blood after mesenteric IR. We found IR-induced significant elevations in plasma ALT and ALP, however as in the other parameters, elimination of RAGE did not significantly alter these elevations (Figures 4A,B). Moreover, HMGB-1 and IL-6 concentrations in the plasma were significantly increased following mesenteric IR. However, these two pro-inflammatory proteins were not altered in RAGE^−/−^ mice after IR (Figures 4C,D).

## Discussion

In this study, we aimed to determine the role of RAGE in the progression of mesenteric IR-induced tissue injury and inflammation. Our hypothesis was that RAGE would contribute to the pathology associated with reperfusion injury, in line with its known pro-inflammatory role, and its documented role in propagating IR injuries in other organs (10–13). We first identified that ligands for RAGE, HMGB-1 and C3a, in the circulation were elevated after 30 min ischemia followed by 150 min reperfusion in the intestine, indicating that enhanced RAGE-signaling was feasibly increased after mesenteric IR. We then used RAGE^−/−^ mice to determine the effect of RAGE absence in intestinal IR injury. Interestingly, we found that all local and remote injury parameters measured including mucosal injury, intestinal edema and neutrophil infiltration, neutrophil mobilization and pro-inflammatory cytokine production, and liver injury markers were not altered by a lack of RAGE-signaling. Overall, these results clearly demonstrate that RAGE is not a critical receptor in the pathogenesis of intestinal IR-induced local injury, systemic inflammation and remote organ damage.

Mesenteric IR accounts for a high mortality rate in the hospital setting, and there is no adequate therapy. A severe pro-inflammatory response is seen upon reperfusion of the ischemic intestine, and leads to tissue destruction and remote organ failure. Thus, numerous therapies have been proposed to treat intestinal IR injury, particularly those targeting the innate immune system responding to alarmins produced by the ischemic tissue. RAGE is a key innate immune receptor that can interact with multiple and diverse ligands, many of which are produced following tissue ischemia, and has been reported to participate in a number of acute and chronic inflammatory diseases through downstream signaling mechanisms of immune and inflammatory responses (25–27). Indeed, in the setting of IR, several studies have described the pathogenic contribution of RAGE in injuries of the liver, lung, heart, and brain (10–13, 28).

One of the key ligands for RAGE is the secreted alarmin/DAMP HMGB-1, which has been clearly documented to mediate pro-inflammatory effects when activating this receptor (7, 29), and is suggested to be a key therapeutic target to reduce tissue injury (11, 12, 30). In our mesenteric IR model, we demonstrated that the levels of HMGB-1 were significantly upregulated following IR. We also showed that another ligand for RAGE, the innate immune complement factor C3a (31), was similarly elevated, indicating the potential for multiple ligands interacting with RAGE, which is expressed on many pro-inflammatory cells involved in IR, including within the intestine (32). Despite this potential for enhanced RAGE activation and pro-inflammatory induction, eliminating RAGE signaling in RAGE^−/−^ mice did not protect local mucosal injury, neutrophil influx and vascular leakage, as well as remote organ liver damage caused by mesenteric IR. Interestingly, Dessing et al. (33) similarly showed that RAGE deficiency does not affect renal injury and function after renal IR, despite RAGE ligands HMGB-1 and S100B being expressed (33). They suggest that RAGE is likely not the primary effector of HMGB-1 in renal IR injury, but rather other innate immune receptors such as the toll-like receptors (TLRs) could be predominating in the pro-inflammatory response. Our study agrees with these findings, and indeed TLRs are known to play major roles in intestinal IR injuries (34).

RAGE–ligand interaction also leads to activation of pro-inflammatory transcription factor nuclear factor kappa B and its downstream target genes, inducing release of pro-inflammatory cytokines such as TNF, IL-1, IL-6, and IL-8 in different cell types (35, 36). We have previously shown IL-6 to be a principal cytokine elevated in the circulation following mesenteric IR (18, 37). In this study, we found, however, that circulating IL-6 (or HMGB1) was not reduced by a lack of RAGE signaling in the IR pathological setting, suggesting a minor role of RAGE in mediating this cytokine release following mucosal injury due to IR. Furthermore, our results also demonstrate that RAGE does not contribute to the intestinal infiltration of neutrophils following mesenteric IR injury. In accordance with these data, leukocyte mobilization following intestinal IR was not altered by the absence of RAGE signaling. Together, this may contribute to the lack of tissue protection in RAGE^−/−^ mice, as neutrophils are known to be key mediators of IR-induced injury (14).

In conclusion, we highlight that deletion of RAGE signaling is not protective against IR-induced mucosal and remote organ injury and systemic inflammation, suggesting that RAGE does not play a role in the pathogenesis of intestinal IR injury in the mouse. Due to the multi-ligand nature of RAGE, in the absence of RAGE, these ligands may favor other pro-inflammatory receptors (such as toll-like receptors), and hence the protective effect of RAGE deletion may be effectively compensated for. Our study thus suggests that blockade of RAGE is not a credible therapeutic target for mesenteric IR injuries.

## Author Contributions

TW conceived the study and together with MW designed the experiments. MW and TG conducted the majority of experiments, with SM providing additional experimental support. MW, TG, and TW wrote the manuscript, with SM providing editorial assistance. All authors approved the final version of the manuscript.

## Conflict of Interest Statement

The authors declare that the research was conducted in the absence of any commercial or financial relationships that could incur a potential conflict of interest.
